# Supplementary material for: A Critical View of the Application of the APEX Software (Aqueous Photochemistry of Environmentally-Occurring Xenobiotics) to Predict Photoreaction Kinetics in Surface Freshwaters
Source: Molecules. 2019 Dec 18;25(1):9. doi: 10.3390/molecules25010009 (PMC7017383; doi:10.3390/molecules25010009)
Supplement: Supplementary file 1 [file molecules-25-00009-s001.zip › 3.2.4_gcc-4.4.0.zip!/3.2.4_gcc-4.4.0/3.2.4_gcc-4.4.0/doc/octave/html/interpreter/Formatted-Input.html]

Formatted Input - Untitled


Next: Input Conversion Syntax,
Previous: Other Output Conversions,
Up: C-Style I/O Functions


---

#### 14.2.11 Formatted Input

Octave provides the `scanf`, `fscanf`, and `sscanf`
functions to read formatted input. There are two forms of each of these
functions. One can be used to extract vectors of data from a file, and
the other is more `C-like'.

— Built-in Function: [val, count] = **fscanf** (fid, template, size)  
— Built-in Function: [v1, v2, ..., count] = **fscanf** (fid, template, "C")  
> In the first form, read from fid according to template,
> returning the result in the matrix val.
>
> The optional argument size specifies the amount of data to read
> and may be one of
>
> `Inf`: Read as much as possible, returning a column vector. nr: Read up to nr elements, returning a column vector. `[`nr`, Inf]`: Read as much as possible, returning a matrix with nr rows. If the number of elements read is not an exact multiple of nr, the last column is padded with zeros. `[`nr`,` nc`]`: Read up to nr `*` nc elements, returning a matrix with nr rows. If the number of elements read is not an exact multiple of nr, the last column is padded with zeros.
>
> If size is omitted, a value of `Inf` is assumed.
>
> A string is returned if template specifies only character
> conversions.
>
> The number of items successfully read is returned in count.
>
> In the second form, read from fid according to template,
> with each conversion specifier in template corresponding to a
> single scalar return value. This form is more `C-like', and also
> compatible with previous versions of Octave. The number of successful
> conversions is returned in count
>
>
>
>
> **See also:** scanf, sscanf, fread, fprintf, fgets, fputs.

— Built-in Function: [val, count] = **scanf** (template, size)  
— Built-in Function: [v1, v2, ..., count]] = **scanf** (template, "C")  
> This is equivalent to calling `fscanf` with fid = `stdin`.
>
> It is currently not useful to call `scanf` in interactive
> programs.
>
>
>
> **See also:** fscanf, sscanf, printf.

— Built-in Function: [val, count] = **sscanf** (string, template, size)  
— Built-in Function: [v1, v2, ..., count] = **sscanf** (string, template, "C")  
> This is like `fscanf`, except that the characters are taken from the
> string string instead of from a stream. Reaching the end of the
> string is treated as an end-of-file condition.
>
>
>
> **See also:** fscanf, scanf, sprintf.

Calls to `scanf` are superficially similar to calls to
`printf` in that arbitrary arguments are read under the control of
a template string. While the syntax of the conversion specifications in
the template is very similar to that for `printf`, the
interpretation of the template is oriented more towards free-format
input and simple pattern matching, rather than fixed-field formatting.
For example, most `scanf` conversions skip over any amount of
“white space” (including spaces, tabs, and newlines) in the input
file, and there is no concept of precision for the numeric input
conversions as there is for the corresponding output conversions.
Ordinarily, non-whitespace characters in the template are expected to
match characters in the input stream exactly.
When a matching failure occurs, `scanf` returns immediately,
leaving the first non-matching character as the next character to be
read from the stream, and `scanf` returns all the items that were
successfully converted.
The formatted input functions are not used as frequently as the
formatted output functions. Partly, this is because it takes some care
to use them properly. Another reason is that it is difficult to recover
from a matching error.
